# OA34 Juvenile Sjogren’s Syndrome: the need for validated paediatric diagnostic criteria. A retrospective review from a tertiary rheumatology centre

**DOI:** 10.1093/rap/rkac066.034

**Published:** 2022-09-28

**Authors:** Charlene Foley, Dearbhla McKenna, Kathy Gallagher, Coziana Ciurtin, Muthana Al Obaidi

**Affiliations:** Great Ormond Street Hospital, London, United Kingdom; Great Ormond Street Hospital, London, United Kingdom; Great Ormond Street Hospital, London, United Kingdom; Centre for Adolescent Rheumatology Versus Arthritis Department of Medicine, University College London (UCL), London, United Kingdom; Great Ormond Street Hospital, London, United Kingdom; NIHR Biomedical Research Centre GOS Institute of Child Health, GOSH NHS Foundation Trust, London, United Kingdom

## Abstract

**Introduction/Background:**

Sjögren’s syndrome (SS) is a chronic multi-system autoimmune disease, characterised by inflammation of the exocrine glands, predominantly salivary and lacrimal glands resulting in xerostomia and xerophthalmia.

Juvenile-onset-SS (jSS) is rare; however, it is probably under-recognised and under-diagnosed. There are no studies reporting accurate incidence or prevalence of jSS.

**Description/Method:**

Diagnosing jSS can be challenging. The cardinal signs and symptoms are non-specific. There is no gold standard biomarker-of-disease. Between 1965–2002 eleven-diagnostic criteria-sets were developed, none of which have gained universal acceptance or been validated in a paediatric-population. Until recently, the most widely used criteria were those developed by the American-European-Consensus-Group (AECG).

It remains well recognised that international consensus on classification is important for standardisation, particularly in relation to research and monitoring treatment outcomes. Subsequently, the 2016 ACR/EULAR classification criteria for SS were developed. However, there remains a paucity of validated criteria for diagnosis of jSS.

Paediatric focussed criteria are required as features of SS in children differ from those in adults. Children experience less dryness and more frequently experience systemic symptoms and parotid enlargement. Applying adult criteria to a paediatric population may lead to mis- and/or under-diagnosis. Bartunkova et al. (1999) proposed paediatric criteria for diagnosis of jSS. These have not been validated.

Objectives

To describe the clinical, laboratory and imaging characteristics of children diagnosed with jSS at Great Ormond Street Hospital (GOSH) over a ten-year period

To apply AECG and the proposed paediatric criteria to our cohort and report percentage fulfilling each criteria

Methods

Retrospective chart review of children diagnosed with jSS from January 2012 - January 2022 at GOSH. Demographic, clinical, laboratory and imaging findings were documented. Features at presentation were compared to the AECG and proposed paediatric SS diagnostic criteria. Percentage of the cohort fulfilling diagnostic criteria for each was calculated.

**Discussion/Results:**

Twenty-eight cases of jSS were diagnosed from January 2012 - January 2022, 22 female (79%). Referring clinicians to Paediatric Rheumatology included Paediatricians (n = 21,75%), Ophthalmologists (n = 1,3.6%), Rheumatologists (n = 1,3.6%), Dermatologists (n = 1, 3.6%), Nephrologists (n = 2,7.1%) and Oral surgeons (n = 2,7.1%). Main reasons for referral included facial swelling, lymphadenopathy, history of rash, constitutional symptoms, and positive investigation findings suggestive of jSS i.e. autoantibody profile and US salivary glands. Median age at diagnosis was 11 years (3.4-14.8).

The most common presenting feature was facial swelling (n-18,64%), followed by lethargy (n = 12,43%) and rash (n = 11,39%). Reports of dry eyes (n = 7, 25%) and mouth (n = 9, 32%) were reported less frequently. Laboratory features identified included anaemia, elevated amylase and ESR, positive ANA and Ro, and hyper IgG. All children who had an USS of their salivary glands had features consistent with a diagnosis of jSS (n = 24,86%). Eleven children had salivary gland biopsy, ten (91%) of which provided tissue confirmation of the jSS diagnosis. A total of 8/28 (28.6%) cases satisfied the AECG criteria; 22/28 (78.6%) satisfied the proposed paediatric SS diagnostic criteria.

**Key learning points/Conclusion:**

Conclusion

A low percentage of our cohort fulfilled the AECG diagnostic criteria, therefore we would suggest application of adult criteria for diagnosis of jSS maybe unhelpful. Inclusion of recurrent parotitis and additional laboratory features of the in the proposed paediatric criteria may increase sensitivity. Our cohort highlights the need for validated jSS diagnostic criteria as paediatric and adult presentations of SS can differ.

